# Burden on the burdened: tuberculosis among Scheduled Tribes and non-Scheduled Tribes in constitutionally protected Scheduled and non-Scheduled areas of India

**DOI:** 10.1186/s40249-025-01375-9

**Published:** 2025-11-03

**Authors:** Nishikant Singh, Sudheer Kumar Shukla, Ritam Dubey, Pratheeba John, Rituparna Sengupta, Ritesh Ranjan Pushkar, Navin Singh, Prince Chugh, Nishant Yadav, Rajeev Sadanandan

**Affiliations:** 1 Health Systems Transformation Platform (HSTP), New Delhi, India; 2 Independent Researcher, New Delhi, India; 3 Directorate of Census Operations Odisha, Office of the Registrar General of India, Bhubaneswar, India; 4 King George’s Medical University, Lucknow, India; 5 Jawaharlal Nehru University, New Delhi, India

**Keywords:** Tuberculosis burden, Scheduled Tribes, Indigenous population, Indian constitution, National Family Health Survey, India

## Abstract

**Background:**

India accounts for over a quarter of the global tuberculosis (TB) burden. Among the most affected are India’s Scheduled Tribes (STs) communities, experiencing a disproportionately higher TB prevalence compared to non-STs. Encouragingly, two successive rounds of National Family Health Survey (NFHS) showed the declined trend in overall TB prevalence in India, the rate of decline was markedly slower among STs, signalling that national gains have not translated into equitable progress. This study examines the point prevalence of TB and its determinants among STs and non-STs populations in constitutionally protected Scheduled and Non-Scheduled areas of India.

**Methods:**

We analysed data from 2,077,924 individuals aged 15 and above from NFHS-5 (2019–2021) in India. Districts were stratified into: (1) Scheduled Area districts (with protections under Schedules V/VI), (2) non-Scheduled districts with > 60% STs, and (3) non-Scheduled districts with < 60% STs. We estimated TB point prevalence per 100,000 among STs and non-STs across these categories and examined associated socio-demographic, environmental, and behavioural factors. Multivariable logistic regression models assessed the adjusted odds of TB.

**Results:**

STs experienced significantly higher TB prevalence (416/100,000) than non-STs (277/100,000). This disparity persisted across all district categories. STs in Scheduled area districts had the lowest TB prevalence (330 per 100,000), while non-Scheduled districts with > 60% STs populations had the highest prevalence (608 per 100,000). Tribal identity remained an independent risk factor for TB [adjusted odd ratio (*aOR*) = 1.47; 95% confidence internal (*CI*) 1.38 −1.56], even after adjusting for covariates. Additional risk factors included older age, male sex, low household wealth, adverse household environments, tobacco and alcohol consumption, and hypertension and diabetes.

**Conclusions:**

Tribal communities continue to shoulder a disproportionate TB burden, reflecting deep-rooted social and structural inequities. While constitutional protections in Scheduled Areas appear to offer some safeguards, disparities between STs and non-STs remain stark. Our findings serve as evidence and a call to action to ensure that tribal communities are at the forefront of TB control initiatives, so that the burden of TB is no longer borne disproportionately by those already burdened by socio-economic disadvantage.

**Graphical Abstract:**

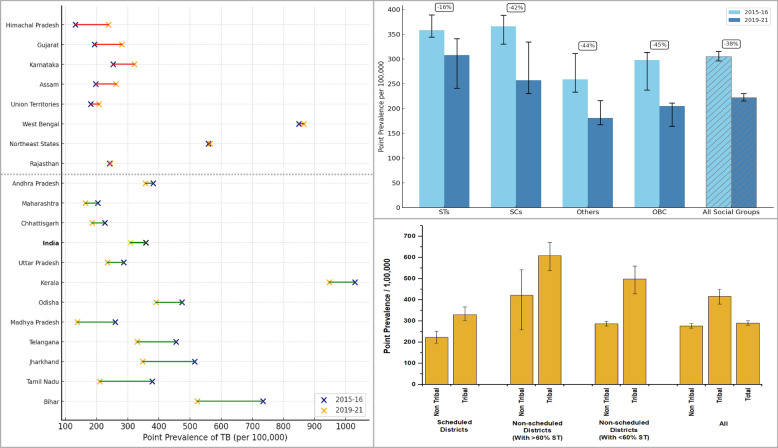

**Supplementary Information:**

The online version contains supplementary material available at 10.1186/s40249-025-01375-9.

## Background

Tuberculosis (TB), one of the oldest known infectious diseases, caused by a bacterium, *Mycobacterium tuberculosis* and usually spreads through respiratory droplets/droplet nuclei in the air, continues to be the leading cause of death from a single infectious agent, claiming approximately 3500 lives per day globally in 2023 [1]. TB remains one of the main health concerns affecting indigenous populations worldwide [2]. Globally, TB is a disease of poverty and is concentrated among populations marginalised from access to healthcare and other social services [3]. Although the estimated 476 million indigenous people worldwide represent less than 5% of the global population, they account for approximately 15% of the world’s poorest [4]. Evidence from countries with available surveillance data indicates that indigenous populations bear a disproportionately high burden of tuberculosis, highlighting the structural and social inequities they continue to face [2, 3, 5]. The heightened risk for TB among indigenous populations reflecting the historic and ongoing displacement and exclusion from access to healthcare and other services [3, 6].

Each year, more than 10 million people contract TB, with the numbers increasing since 2021. Majority of these cases remains concentrated in the WHO regions of South-East Asia (45%), Africa (24%) and the Western Pacific (17%). In 2023, 30 countries accounted for 87% of the global TB burden, with 5 countries contributing to over 56% of the total burden [7]. India remains the country with the highest TB burden, responsible for 26% of global cases, followed by Indonesia (10%) and China (6.8%) in 2023. Along with 2.7 million new cases of TB in 2023, India accounted for 17% of the global deaths due to TB [7, 8]. India’s socio-economic and demographic diversity contributes to notable disparities in prevalence of TB across the country with the marginalized populations particularly the tribal communities facing disproportionate risks. Accounting for 8.6% of the country’s population (110.4 million) [9], the Scheduled Tribes (STs) in India exhibited a higher prevalence of TB at 703 per 100,000 compared to the national average of 316 per 100,000 population [10, 11]. Gaping disparities in health status of the tribals and general population are observed, with the former facing a higher burden of morbidity and mortality across diverse groups. A systematic review by Thomas et al. revealed variations in TB incidence among different tribal groups in Madhya Pradesh ranging from 146 per 100,000 among *Baigas* to 1518 per 100,000 among the *Sahariyas* [10].

**Fig. 1:**
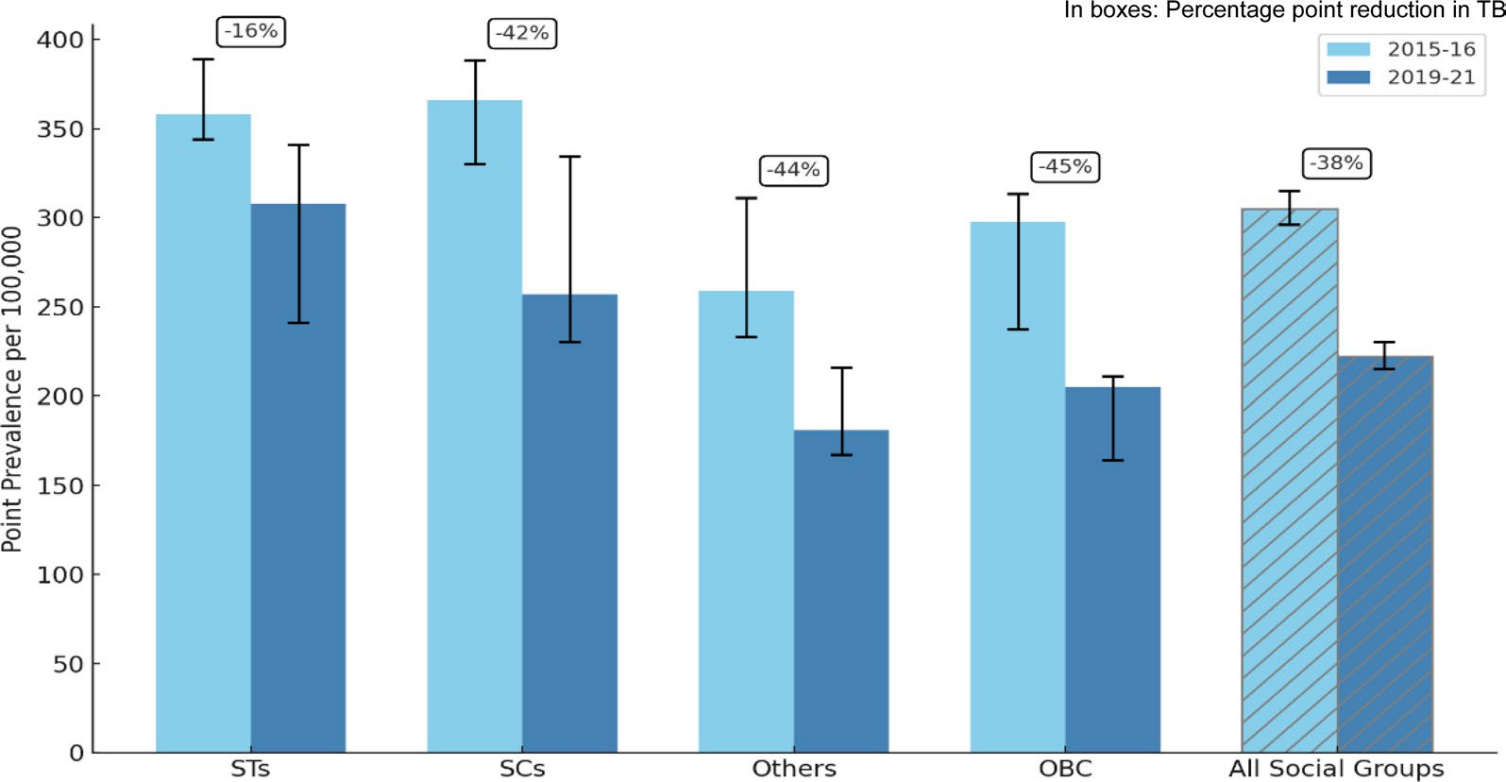
Point prevalence of tuberculosis per 100,000 persons aged 15 and above, and percentage point reduction (2015–2016 to 2019–2021) by social groups in India, based on NFHS-4 (2015–2016) and NFHS-5 (2019–2021) national report estimates

**Fig. 2:**
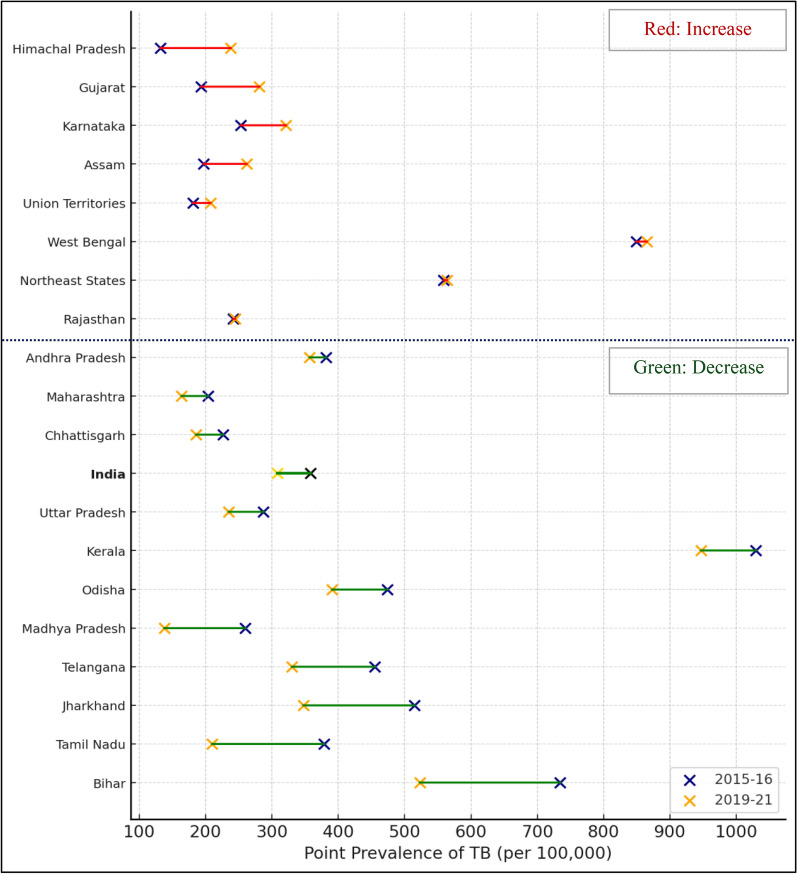
Change in point prevalence of tuberculosis among Schedule Tribes (STs) aged 15 and above by States between 2015–2016 and 2019–2021, based on NFHS-4 (2015–2016) and NFHS-5 (2019–2021) national report estimates

On the brighter side, National Family Health Survey (NFHS) data indicates a downward trend in overall TB prevalence. Figure 1 shows the change in point prevalence of self-reported medically treated TB across major social groups in India, based on NFHS-4 (2015–2016) and NFHS-5 (2019–2021) national reports. Overall, TB prevalence declined from 305 per 100,000 to 222 per 100,000 during this period [12, 13]. A similar decline was observed across all major social groups, but the reduction in TB prevalence among STs was notably smaller than in other social groups. In fact, in several states, TB prevalence among STs increased from NFHS-4 to NFHS-5, as shown in Fig. 2, which draws from national report estimates to contextualise the need for a more granular district-level analysis. These divergent trends indicate that broader TB control efforts have not equitably reached India’s tribal communities, highlighting the need for a more detailed, disaggregated, and focused examination of disparities among STs populations. Multiple interrelated factors contribute to persistently high TB prevalence among STs. Poverty, limited awareness about TB programmes, poor living conditions and weak community participation, shaped in part by experiences of healthcare exclusion, pose significant challenges [3]. Importantly, traditional healing practices, which are culturally rooted and widely respected, may reflect both indigenous knowledge systems and the limited accessibility or acceptability of formal healthcare services [14]. In addition to these, health systems constraints such as irregular availability of medicines in the health facilities in the tribal areas and gaps in service delivery have been reported as the key factors responsible for high prevalence of TB among the STs in India [15, 16]. Each of these factors can hinder TB detection, treatment adherence, and prevention.

Recognizing the disproportionate burden of TB among STs, the National TB Elimination program has prioritized the STs through the Tribal Action Plan since 2005, which aim to improve the care and support services for TB among the STs [9, 17]. To ensure participation in the TB programs, the Government provides a one-time financial incentive of Rs 750 to the notified TB patient residing in the tribal areas [17]. However, despite persistent efforts of the Government, the prevalence of TB remains high among the marginalized STs of the country, with several barriers to its prevention and treatment.

While several studies have examined the prevalence of TB among STs in India, most have primarily concentrated on clinical dimensions. Important social and proximate determinants have not been adequately studied in a nationally representative framework. Moreover, existing research is often limited to a few high-burden states or in regions with predominantly tribal populations. There is no prior district-level comparative analysis of TB burden that stratifies regions by both tribal concentration and their constitutional status regarding tribal self-governance. Under the Constitution of India, certain tribal dominated areas are designated as Scheduled Areas (in Fifth and Sixth Schedules) which grant administrative autonomy and resource protections to tribal communities. How these constitutional safeguards influence the health outcomes (such as TB prevalence) remains unknown, as literature to date has not addressed the interplay between being an individual from a STs and living in a Scheduled Area. Given the recent findings indicating rising TB prevalence among STs in some states despite an overall national decline, there is an urgent need for a more granular analysis to identify where and why STs continue to bear a higher burden.

The present study therefore aims to quantify and compare the burden of TB among STs and non-STs populations across different types of districts in India, defined by STs concentration and constitutional status. Using the latest NFHS-5 data, we conduct what is, to our knowledge, the first national district-level comparative analysis of TB that differentiates between Scheduled Area districts versus non-Scheduled districts (further categorized into those with high vs. low tribal population share). Beyond assessing TB prevalence, the study examines how proximate determinants such as housing conditions, access to amenities, and behavioral risk factors interact with the disease burden. By adopting an equity-focused approach, this research seeks to provide a comprehensive understanding of the structural and social factors driving TB disparities in tribal communities.

## Method

### Data source

We used data from the fifth round of the National Family Health Survey (NFHS-5), India’s implementation of the global Demographic and Health Survey (DHS) program. NFHS-5, the largest household health survey conducted in India to date, was commissioned by the Ministry of Health and Family Welfare, Government of India, and carried out in two phases: Phase I (17 June 2019 to 30 January 2020) covered 17 states and 5 union territories (UTs), while Phase II (2 January 2020 to 30 April 2021) covered 11 states and 3 UTs. The survey was implemented by 17 field agencies and collected data from 636,699 households, 724,115 women, and 101,839 men aged 15–49 years. Household response rates exceeded 95% across all states and UTs, except Chandigarh (88%) and Madhya Pradesh (94%). NFHS-5 followed a multistage stratified sampling design. In the first stage, states were divided into regions based on political or geographical boundaries and further stratified by urban and rural residence. Enumeration areas were then selected using probability proportional to size sampling from the most recent census. In the second stage, all households in the selected clusters were listed, and approximately 25 households per cluster were systematically sampled with equal probability. Detailed information on the sampling design and survey instruments is available in the NFHS-5 report [13].

### Study population

The NFHS-5 person-level dataset includes 2,843,917 individuals. For data analysis we considered all usual residents aged 15 years and above, who constituted approximately 73% of the total sample. Individuals below 15 years of age were excluded, as their TB risk profile and disease progression differ substantially from that of adults, requiring distinct clinical and epidemiological considerations [18]. Our final analytic sample size was 2,077,924 individuals after excluding cases with missing data on key variables.

### Outcome variable

Information on TB was extracted from the household schedule of the NFHS-5 survey, wherein an eligible female respondent provided data on all usual household members and any visitors who stayed in the household the night before the survey. Respondents were asked a single screening question: “Does any usual resident of your household suffer from tuberculosis?” If the answer was affirmative, a follow-up question inquired whether the affected individual had sought medical treatment. For the purpose of this study, we included only those individuals who were reported to have suffered from TB and had received medical treatment.

### Socioeconomic, demographic, and proximate covariates

Patterns and determinants for TB were explored via selected demographic and socioeconomic factors, as well as relevant proximate covariates, for both STs and non-STs populations. These variables were chosen based on current research from India and other low- and middle-income countries concerning the determinants of TB (Supplementary Table 1). We ensured that the variables included were consistently available for individuals aged 15 years and older within the study population.

### Scheduled and non-Scheduled areas

The concept of Scheduled Area existed even before independence which were governed by special arrangements that protect and promote the interests of STs in India. It is retained in form of Article 244(1) and 244(2) of the Constitution of India, that are designated as Scheduled Areas in the Fifth and Sixth Schedules of the Constitution, respectively. Article 244(1) provides provisions for the administration of Scheduled Area mentioned in Fifth Schedule in states of Andhra Pradesh, Telangana, Odisha, Jharkhand, Chhattisgarh, Madhya Pradesh, Rajasthan, Gujarat, Maharashtra and Himachal Pradesh. While Article 244(2) enlists special provisions for the administration of Scheduled Area mentioned in Sixth Schedule in states of Assam, Meghalaya, Tripura and Mizoram. For the purpose of this study, Scheduled Areas covered under 5th and 6th schedule of the Constitution of India are taken as a unit as they have somewhat similar provision for autonomy and self-governance to the tribal communities. However, Scheduled Areas do not always align perfectly with district boundaries—often only certain blocks or villages within a district are notified as Scheduled Area. For our analysis, we defined a “Scheduled Area district” as any district that is wholly or partially designated as a Scheduled Area under either the Fifth or Sixth Schedule. This was done as a pragmatic approach since NFHS data could not be disaggregated below the district level to isolate only the exact sub-district Scheduled territories. Thus, if any part of a district is a Scheduled Area, we treated the entire district as belonging to the Scheduled Area category.

The remaining districts (those with no Fifth/Sixth Schedule area) were further subdivided by the concentration of STs. We calculated the percentage of the district’s population that belonged to STs using NFHS-5. Districts without Scheduled Area status was classified into two groups: high tribal concentration (more than 60% of the population is STs) and low tribal concentration (less than 60% STs). The 60% threshold [19] was chosen to distinguish districts where STs form a majority or very large minority of the population (potentially reflecting different social dynamics) from those where STs are a smaller minority. This yielded three mutually exclusive district categories for comparison (Table 1):

- Scheduled Area districts (under Schedule V or VI; autonomy protections in place),
- Non-Scheduled districts, > 60% STs (no special protections, but STs form a majority/high share of population),
- Non-Scheduled districts, < 60% STs (no special protections, STs in minority).

**Table 1:** Distribution of Scheduled and Non-Scheduled district categories selected for the study, NFHS-5 (2019–21)

State/UT	Districts
Scheduled districts (136 districts)	
Andhra Pradesh	Srikakulam, Vizianagaram, Visakhapatnam, East Godavari, West Godavari, Krishna, Guntur, Prakasam, Sri Potti Sriramulu Nellore, Y.S.R., Kurnool, Anantapur, Chittoor
Assam	Kokrajhar, Dima Hasao, Chirang, Baksa, Udalguri, Karbi Anglong
Chhattisgarh	Koriya, Jashpur, Raigarh, Korba, Rajnandgaon, Dhamtari, Uttar Bastar Kanker, Narayanpur, Bijapur, Bastar, Bilaspur, Dantewada, Raipur, Surguja
Gujarat	Banas Kantha, Dohad, Narmada, Bharuch, The Dangs, Navsari, Valsad, Surat, Tapi, Panch Mahals, Sabar Kantha, Vadodara
Himachal Pradesh	Chamba, Lahul & Spiti, Kinnaur
Jharkhand	Garhwa, Godda, Sahibganj, Pakur, Lohardaga, Purbi Singhbhum, Palamu, Latehar, Dumka, Jamtara, Ranchi, Khunti, Gumla, Simdega, Pashchimi Singhbhum, Saraikela-Kharsawan
Madhya Pradesh	Sheopur, Umaria, Ratlam, Dewas, Dhar, Khargone (West Nimar), Barwani, Betul, Harda, Hoshangabad, Katni, Jabalpur, Dindori, Mandla, Chhindwara, Seoni, Balaghat, Shahdol, Anuppur, Sidhi, Singrauli, Jhabua, Alirajpur, Khandwa (East Nimar)
Maharashtra	Nandurbar, Dhule, Jalgaon, Amravati, Gadchiroli, Chandrapur, Yavatmal, Nanded, Nashik, Pune, Ahmadnagar, Thane
Meghalaya	South Garo Hills, Ribhoi, East Khasi Hills, East Garo Hills, East Jantia Hills, South West Khasi Hill, West Garo Hills, West Jaintia Hills
Mizoram	Lawngtlai, Saiha
Odisha	Jharsuguda, Sambalpur, Sundargarh, Kendujhar, Mayurbhanj, Baleshwar, Dhenkanal, Gajapati, Kandhamal, Kalahandi, Rayagada, Nabarangapur, Koraput, Malkangiri
Rajasthan	Sirohi, Pali, Rajsamand, Dungarpur, Banswara, Chittaurgarh, Udaipur, Pratapgarh
Tripura	Dhalai, North Tripura, South Tripura, West Tripura
Non-scheduled districts with > 60% of STs (51 districts)	
Andaman & Nicobar Islands (UT)	Nicobars
Arunachal Pradesh	Tawang, West Kameng, East Kameng, Papum Pare, Upper Subansiri, Upper Siang, Lower Subansiri, Dibang Valley, Lower Dibang Valley, Anjaw, East Siang, Kra Daadi, Kurung Kumey, Longding, Siang, Tirap, West Siang
Assam	West Karbi Anglong
Chhattisgarh	Balrampur, Kodagaon, Sukma
Gujarat	Chhota Udaipur
Ladakh (UT)	Kargil
Lakshadweep (UT)	Lakshadweep
Manipur	Senapati, Tamenglong, Churachandpur, Ukhrul, Chandel
Meghalaya	North Garo Hills, South West Garo Hills, West Khasi Hills
Mizoram	Mamit, Kolasib, Aizawl, Champhai, Serchhip, Lunglei
Nagaland	Mon, Mokokchung, Zunheboto, Wokha, Dimapur, Phek, Tuensang, Longleng, Kiphire, Kohima, Peren
Sikkim	North District
Non-scheduled districts with < 60% of STs (520 districts)	
Andaman & Nicobar Islands (UT)	North & Middle Andaman, South Andaman
Arunachal Pradesh	Changlang, Lohit, Namsai
Assam	Goalpara, Barpeta, Morigaon, Lakhimpur, Dhemaji, Tinsukia, Dibrugarh, Golaghat, Cachar, Karimganj, Hailakandi, Bongaigaon, Kamrup, Kamrup Metropolitan, Nalbari, Darrang, Biswanath, Charaideo, Dhubri, Hojai, Jorhat, Majuli, Nagaon, Sivasagar, Sonitpur, South Salmara Mankachar
Bihar	Pashchim Champaran, Purba Champaran, Sheohar, Sitamarhi, Madhubani, Supaul, Araria, Kishanganj, Purnia, Katihar, Madhepura, Saharsa, Darbhanga, Muzaffarpur, Gopalganj, Siwan, Saran, Vaishali, Samastipur, Begusarai, Khagaria, Bhagalpur, Banka, Munger, Lakhisarai, Sheikhpura, Nalanda, Patna, Bhojpur, Buxar, Kaimur (Bhabua), Rohtas, Aurangabad, Gaya, Nawada, Jamui, Jehanabad, Arwal
Chandigarh	Chandigarh
Chhattisgarh	Janjgir-Champa, Kabeerdham, Mahasamund, Balod, Baloda Bazar, Bemetara, Durg, Gariyaband, Mungeli, Surajpur
Dadra & Nagar Haveli and Daman & Diu (UT)	Diu, Daman, Dadra & Nagar Haveli
Goa	North Goa, South Goa
Gujarat	Kachchh, Patan, Mahesana, Gandhinagar, Porbandar, Amreli, Anand, Ahmedabad, Aravalli, Bhavnagar, Botad, Devbhumi Dwarka, Gir Somnath, Jamnagar, Junagadh, Kheda, Mahisagar, Morbi, Rajkot, Surendranagar
Haryana	Panchkula, Ambala, Yamunanagar, Kurukshetra, Kaithal, Karnal, Panipat, Sonipat, Jind, Fatehabad, Sirsa, Hisar, Rohtak, Jhajjar, Mahendragarh, Rewari, Gurgaon, Mewat (Nuh), Faridabad, Palwal, Bhiwani, Charkhi Dadri
Himachal Pradesh	Kangra, Kullu, Mandi, Hamirpur, Una, Bilaspur, Solan, Sirmaur, Shimla
Jammu & Kashmir	Kupwara, Badgam, Punch, Rajouri, Kathua, Baramula, Bandipore, Srinagar, Ganderbal, Pulwama, Shupiyan, Anantnag, Kulgam, Doda, Ramban, Kishtwar, Udhampur, Reasi, Jammu, Samba
Jharkhand	Chatra, Kodarma, Giridih, Deoghar, Dhanbad, Bokaro, Hazaribagh, Ramgarh
Karnataka	Belgaum, Bagalkot, Bijapur, Bidar, Raichur, Koppal, Gadag, Dharwad, Uttara Kannada, Haveri, Bellary, Chitradurga, Davanagere, Shimoga, Udupi, Chikmagalur, Tumkur, Bangalore, Mandya, Hassan, Dakshina Kannada, Kodagu, Mysore, Chamarajanagar, Gulbarga, Yadgir, Kolar, Chikkaballapura, Bangalore Rural, Ramanagara
Kerala	Kasaragod, Kannur, Wayanad, Kozhikode, Malappuram, Palakkad, Thrissur, Ernakulam, Idukki, Kottayam, Alappuzha, Pathanamthitta, Kollam, Thiruvananthapuram
Ladakh (UT)	Leh (Ladakh)
Madhya Pradesh	Morena, Bhind, Gwalior, Datia, Shivpuri, Tikamgarh, Chhatarpur, Panna, Sagar, Damoh, Satna, Rewa, Neemuch, Mandsaur, Ujjain, Indore, Rajgarh, Vidisha, Bhopal, Sehore, Raisen, Narsimhapur, Guna, Ashoknagar, Burhanpur, Agar Malwa, Shajapur
Maharashtra	Buldana, Akola, Washim, Wardha, Nagpur, Bhandara, Gondiya, Hingoli, Parbhani, Jalna, Aurangabad, Mumbai Suburban, Mumbai, Raigarh, Bid, Latur, Osmanabad, Solapur, Satara, Ratnagiri, Sindhudurg, Kolhapur, Sangli, Palghar
Manipur	Bishnupur, Thoubal, Imphal West, Imphal East
NCT of Delhi	Central, East, New Delhi, North, North East, North West, Shahdara, South, South East, South West, West
Odisha	Bargarh, Debagarh, Bhadrak, Kendrapara, Jagatsinghapur, Cuttack, Jajapur, Anugul, Nayagarh, Khordha, Puri, Ganjam, Baudh, Subarnapur, Balangir, Nuapada
Puducherry (UT)	Yanam, Puducherry, Mahe, Karaikal
Punjab	Kapurthala, Jalandhar, Hoshiarpur, Shahid Bhagat Singh Nagar, Fatehgarh Sahib, Ludhiana, Moga, Muktsar, Faridkot, Bathinda, Mansa, Patiala, Amritsar, Tarn Taran, Rupnagar, Sahibzada Ajit Singh Nagar, Sangrur, Barnala, Fazilka, Firozpur, Gurdaspur, Pathankot
Rajasthan	Ganganagar, Hanumangarh, Bikaner, Churu, Jhunjhunun, Alwar, Bharatpur, Dhaulpur, Karauli, Sawai Madhopur, Dausa, Jaipur, Sikar, Nagaur, Jodhpur, Jaisalmer, Barmer, Jalor, Ajmer, Tonk, Bundi, Bhilwara, Kota, Baran, Jhalawar
Sikkim	West District, South District, East District
Tamil Nadu	Thiruvallur, Chennai, Kancheepuram, Vellore, Tiruvannamalai, Viluppuram, Salem, Namakkal, Erode, The Nilgiris, Dindigul, Karur, Tiruchirappalli, Perambalur, Ariyalur, Cuddalore, Nagapattinam, Thiruvarur, Thanjavur, Pudukkottai, Sivaganga, Madurai, Theni, Virudhunagar, Ramanathapuram, Thoothukkudi, Tirunelveli, Kanniyakumari, Dharmapuri, Krishnagiri, Coimbatore, Tiruppur
Telangana	Adilabad, Bhadradri Kothagudem, Hyderabad, Jagitial, Jangaon, Jayashankar Bhupalapally, Jogulamba Gadwal, Kamareddy, Karimnagar, Khammam, Komaram Bheem Asifabad, Mahabubabad, Mahabubnagar, Mancherial, Medak, Medchal-Malkajgiri, Nagarkurnool, Nalgonda, Nirmal, Nizamabad, Peddapalli, Rajanna Sircilla, Ranga Reddy, Sangareddy, Siddipet, Suryapet, Vikarabad, Wanaparthy, Warangal Rural, Warangal Urban, Yadadri Bhuvanagiri
Tripura	Gomati, Khowai, Sepahijala, Unakoti
Uttar Pradesh	Saharanpur, Bijnor, Rampur, Jyotiba Phule Nagar, Meerut, Baghpat, Gautam Buddha Nagar, Bulandshahr, Aligarh, Mahamaya Nagar, Mathura, Agra, Firozabad, Mainpuri, Bareilly, Pilibhit, Shahjahanpur, Kheri, Sitapur, Hardoi, Unnao, Lucknow, Farrukhabad, Kannauj, Etawah, Auraiya, Kanpur Dehat, Kanpur Nagar, Jalaun, Jhansi, Lalitpur, Hamirpur, Mahoba, Banda, Chitrakoot, Fatehpur, Pratapgarh, Kaushambi, Allahabad, Bara Banki, Faizabad, Ambedkar Nagar, Bahraich, Shrawasti, Balrampur, Gonda, Siddharthnagar, Basti, Sant Kabir Nagar, Mahrajganj, Gorakhpur, Kushinagar, Deoria, Azamgarh, Mau, Ballia, Jaunpur, Ghazipur, Chandauli, Varanasi, Sant Ravidas Nagar (Bhadohi), Mirzapur, Sonbhadra, Etah, Kanshiram Nagar, Amethi, Budaun, Ghaziabad, Hapur, Moradabad, Muzaffarnagar, Rae Bareli, Sambhal, Shamli, Sultanpur
Uttarakhand	Uttarkashi, Chamoli, Rudraprayag, Tehri Garhwal, Dehradun, Garhwal (Pauri), Pithoragarh, Bageshwar, Almora, Champawat, Nainital, Udham Singh Nagar, Haridwar
West Bengal	Darjiling, Jalpaiguri, Koch Bihar, Uttar Dinajpur, Dakshin Dinajpur, Maldah, Murshidabad, Birbhum, Nadia, North Twenty Four Parganas, Hugli, Bankura, Puruliya, Haora, Kolkata, South Twenty Four Parganas, Paschim Medinipur, Purba Medinipur, Paschim Barddhaman, Purba Barddhaman

The conditions in Scheduled Areas differ considerably from Non-Scheduled Areas, particularly in relation to legal protections, governance structures, and socio-economic contexts. Scheduled Areas are governed by special constitutional provisions under the Fifth and Sixth Schedules, which aim to protect and promote political autonomy through mechanisms like the Tribes Advisory Councils (under Vth Schedule) and Autonomous District Councils (Under VIth Schedule) [19, 20]. These regions are also marked by geographic isolation, higher overlaps with forests, and intense pressures from land acquisition, mining, and conservation laws. These unique features may influence service delivery-sometimes enabling more community-based governance and protection, but often resulting in administrative neglect, resource extraction without consent, and limited infrastructure development [21]. The presence of such divergent structural characteristics provides an important explanatory framework for understanding disparities in service provision between Scheduled and Non-Scheduled Areas, meriting deeper investigation into their causal impacts.

### Statistical analysis

First, we presented the sample distribution using weighted percentages and unweighted counts disaggregated by tribal and non-tribal districts, across the selected background characteristics. As our outcome of interest was self-reported medically treated TB, we initially estimated the point prevalence of TB per 100,000 individuals in India, stratified by key independent variables for STs and non-STs populations separately, as well as for the overall population. We employed the Chi-square test to assess associations between the selected independent variables and the outcome variable. Subsequently, we reported the point prevalence of TB per 100,000 individuals across identified categories of districts, again disaggregated by STs and non-STs status. All prevalence estimates and corresponding 95% confidence intervals (*CI*s) were derived using survey analysis procedures to account for the complex sampling design and applied sampling weights. To examine the factors associated with TB, we conducted multivariate logistic regression analyses, including selected background characteristics as explanatory variables. Results are reported as adjusted odds ratios (a*OR*s) with 95% *CI*s and corresponding *P*-values, representing the likelihood of being reported as having TB.

## Results

### Sample distribution

A total of 2,077,924 individuals aged 15 and above were included in the study, with tribal populations comprising 9.2% of the sample. The dataset covered 136 districts under Schedule V and VI, with 386,262 individuals; 51 districts with > 60% tribal population, including 132,494 individuals; and 520 districts with < 60% tribal population, comprising 1,559,168 individuals (Supplementary Table 2 and Supplementary Table 3). These districts represented 41%, 89%, and 6% of the STs within their respective categories. Females accounted for 51.4% of the total sample, consistently forming the largest share across all district categories. Similarly, individuals aged 15–29 constituted the highest proportion in all district categories.

### Point prevalence of TB

**Fig. 3:**
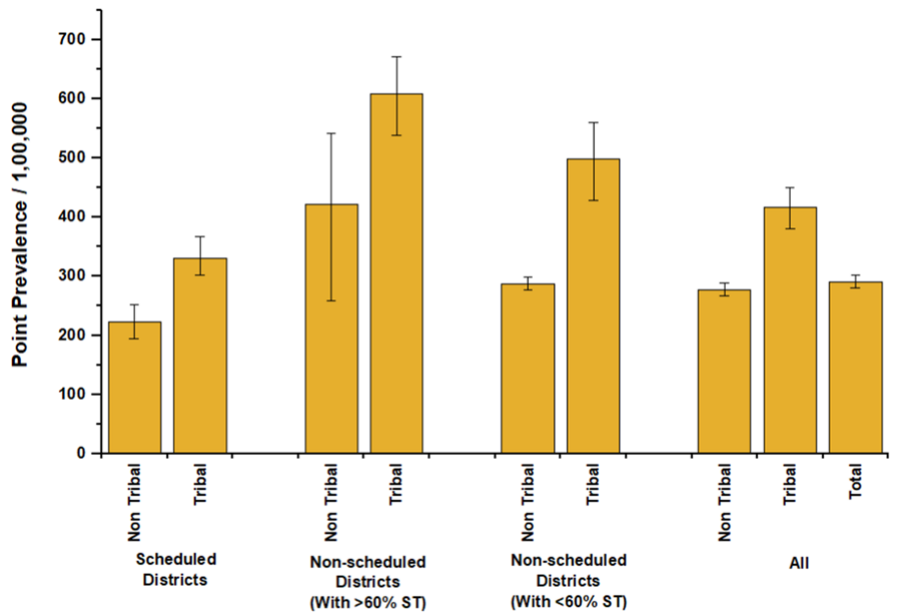
Point prevalence of TB among STs and Non-STs aged 15 and above by different district categories of India, based on unit level data analysis of NFHS-5 (2019–2021). *TB* Tuberculosis, *ST* Schedule Tribe

Across all districts, the overall point prevalence (per 100,000) of TB was 277 (95% *CI* 266–287) for non STs and 416 (95% *CI* 383–452) for STs. In Scheduled area districts marked under Schedule V and VI, the point prevalence per 100,000 persons was 224 (95% *CI* 197–253) for non STs and notably higher at 330 (95% *CI* 294–368) for STs. In non-scheduled districts with more than 60% STs, the point prevalence per 100,000 was 422 (95% *CI* 303–585) among non STs, while tribal populations showed a higher prevalence of 608 (95% *CI* 545–678). In non-scheduled districts with less than 60% STs population, the point prevalence per 100,000 was 287 (95% *CI* 275–298) for non-tribal groups and 498 (95% *CI* 436–568) for STs (Fig. 3).

Among individuals aged 15 and above, TB point prevalence per 100,000 persons was consistently higher among STs across key characteristics (Table 2). STs in the age group 45–59 (626, 95% *CI* 539–726) and 60 + (723, 95% *CI* 610–855) categories had significantly higher prevalence than non-STs (390, 95% *CI* 363–418 and 554, 95% *CI* 519–590, respectively). Tribal males (551, 95% *CI* 497–610) had a higher prevalence than non-tribal males (360, 95% *CI* 343–377). Illiterate STs exhibited a higher prevalence (605, 95% *CI* 539–677) than non-STs (501, 95% *CI* 472–530). Rural tribals (418, 95% *CI* 384–453) had higher prevalence than rural non-tribals (311, 95% *CI* 298–323). Households using unimproved fuel for cooking showed higher prevalence among STs (467, 95% *CI* 426–510) compared to non-STs (366, 95% *CI* 348–384), while those without potable water had a prevalence of 501 (95% *CI* 445–564) among STs, compared to 317 (95% *CI* 302–331) in non- STs. STs with diabetes (552, 95% *CI* 423–719) and hypertension (438, 95% *CI* 368–520) had higher prevalence than non- STs (400, 95% *CI* 364–439 and 300, 95% *CI* 276–325, respectively). Alcohol and tobacco consumption was associated with higher TB prevalence among STs (742, 95% *CI* 635–865) and (657, 95% *CI* 585–737) compared to non- STs (560, 95% *CI* 511–612) and (511, 95% *CI* 482–541) respectively.

**Table 2:** Point prevalence of tuberculosis per 100,000 persons aged 15 and above by demographic, socio–economic and proximate characteristics in India, NFHS-5 (2019–2021)

Ethnicity	Non-STs			STs			Total		
Characteristics	Point prev	95% CI	p value	Point prev	95% CI	p value	Point prev	95% CI	p value
Age			< 0.001			< 0.001			< 0.001
15–29	125	113–138		211	174–255		134	122–146	
30–44	217	199–235		382	324–448		233	215–250	
45–59	390	363–418		626	539–726		411	385–438	
60 +	554	519–590		723	610–855		567	533–602	
Sex			< 0.001			< 0.001			< 0.001
Female	197	184–210		288	250–331		206	193–218	
Male	360	343–377		551	497–610		377	361–394	
Education			< 0.001			< 0.001			< 0.001
Illiterate	501	472–530		605	539–677		515	488–541	
Primary	389	356–423		451	372–545		395	364–427	
Secondary	194	181–207		273	232–321		200	188–213	
Higher	88	73–105		236	147–375		95	80–112	
Wealth quintile			< 0.001			0.007			< 0.001
Poorest	527	494–562		508	456–566		523	494–552	
Poorer	352	326–379		427	363–502		361	337–386	
Middle	255	233–279		285	233–347		257	236–280	
Richer	197	178–218		267	177–402		200	181–221	
Richest	144	126–163		275	146–518		147	129–166	
Place of residence			< 0.001			0.048			< 0.001
Urban	214	196–234		408	303–548		222	203–241	
Rural	311	298–323		418	384–453		324	311–335	
Household size			< 0.001			< 0.001			< 0.001
1–3 members	340	315–366		607	515–714		364	340–389	
4–5 members	247	231–263		371	323–424		259	244–274	
6 & above members	272	255–289		356	312–405		279	263–295	
Persons sleeping per room			< 0.001			0.533			< 0.001
< 3 person	302	288–314		429	389–472		314	301–326	
3–4 person	208	191–227		375	319–439		222	205–240	
> 5 person	186	140–246		363	237–555		199	154–255	
Type of house			< 0.001			< 0.001			< 0.001
Natural	406	378–434		469	421–521		420	396–444	
Rudimentary	354	331–377		422	353–503		359	337–382	
Finished	210	197–223		344	288–410		217	204–230	
Separate kitchen			< 0.001			0.066			< 0.001
Yes	216	203–228		321	282–365		224	212–236	
No	395	368–422		472	408–545		403	378–428	
Type of fuel used for cooking			< 0.001			0.974			< 0.001
Improved	219	206–232		313	258–379		224	211–237	
Unimproved	366	348–384		467	426–510		381	365–398	
Potability of water			< 0.001			0.001			< 0.001
Yes	220	205–236		332	299–369		233	218–247	
No	317	302–331		501	445–564		332	317–345	
Shared toilet			< 0.001			< 0.001			< 0.001
No	237	225–248		344	308–384		245	234–256	
Yes	325	283–371		596	401–883		342	300–388	
Smoking inside the house			< 0.001			< 0.001			< 0.001
Never	231	218–243		349	308–396		240	227–252	
Yes	341	323–359		475	425–529		356	339–373	
Diabetes			< 0.001			< 0.001			< 0.001
No	270	258–282		405	369–444		283	271–295	
Yes	400	364–439		552	423–719		412	376–449	
Hypertension			< 0.001			< 0.001			< 0.001
No	284	270–296		421	381–464		297	284–309	
Yes	300	276–325		438	368–520		312	289–336	
Consumption of alcohol			< 0.001			< 0.001			< 0.001
No	249	238–259		338	307–372		256	246–266	
Yes	560	511–612		742	635–865		592	547–640	
Consumption of tobacco			< 0.001			< 0.001			< 0.001
No	210	199–221		288	256–324		217	206–227	
Yes	511	482–541		657	585–737		531	504–559	
Overall	277	266–287		416	383–452		290	279–300	

*NFHS* National Family Health Survey, *ST* Schedule Tribe

Among scheduled area districts, the point prevalence per 100,000 persons was consistently higher among the STs across most background characteristics compared to non STs. For instance, TB prevalence increased with age in both groups, with STs populations showed higher prevalence in every age category, notably 578 (95% *CI* 458–726) in the 60 + group compared to 415 (95% *CI* 338–509) among non- STs (Table 3). Males had higher prevalence than females in both groups, with tribal males (449, 95% *CI* 392–514) surpassing non-tribal males (300, 95% *CI* 257–350). Illiterate individuals showed the highest prevalence, with STs at 445 (95% *CI* 378–523) compared to non-STs at 420 (95% *CI* 352–500), while STs with higher education (220, 95% *CI* 108–443) also had higher prevalence than non-STs (46, 95% *CI* 25–81). The poorest groups in both populations reported the highest prevalence, with tribal groups in richer quintiles showed higher prevalence than non-tribals. Rural tribal populations (336, 95% *CI* 298–378) had higher prevalence than rural non-tribals (234, 95% *CI* 205–266), and smaller tribal households (1−3 members) showed much higher prevalence (529, 95% *CI* 430–649) than non-tribals (297, 95% *CI* 241–364). Tribals in natural (373, 95% *CI* 323–429) and rudimentary houses (341, 95% *CI* 257–451) exhibit higher prevalence than non-tribals, with households lacking a separate kitchen and using unimproved fuel also showed higher prevalence among tribals (387, 95% *CI* 318–470 and 359, 95% *CI* 317–405, respectively). Risk factors such as smoking, diabetes, hypertension, alcohol, and tobacco consumption are associated with higher prevalence among STs, particularly for diabetes (469, 95% *CI* 349–628) and hypertension (392, 95% *CI* 311–493) compared to non-STs (373, 95% *CI* 290–479 and 240, 95% *CI* 179–320) respectively.

**Table 3:** Point prevalence of TB per 100,000 persons aged 15 and above by demographic, socio–economic and proximate characteristics in different combination of districts with concentration of tribal population in India, NFHS-5 (2019–2021)

Number of districts (N)	Scheduled area districts				Non–scheduled districts							
	Districts marked with Schedule V & VI				Districts with > 60% STs				Districts with < 60% STs			
	136 (386,262)				51 (132,494)				520 (1,559,168)			
Ethnicity	Non-STs		STs		Non-STs		STs		Non-STs		Tribal	
Characteristics	Point prev	95% *CI*	Point prev	95% *CI*	Point prev	95% *CI*	Point prev	95% *CI*	Point prev	95% *CI*	Point prev	95% *CI*
Age												
15–29	83	58–118	147	112–191	167	61–456	298	231–383	133	119–147	280	211–372
30–44	170	128–225	331	267–410	418	241–721	609	486–762	226	207–246	416	315–547
45–59	364	293–451	498	407–609	626	361–1082	747	607–917	395	366–425	767	605–969
60 +	415	338–509	578	458–726	840	406–1730	1197	984–1452	579	540–619	835	637–1094
Sex												
Female	148	120–183	216	177–264	281	150–524	467	395–550	206	192–220	354	284–439
Male	300	257–350	449	392–514	558	380–818	756	654–872	371	353–389	649	549–766
Education												
Illiterate	420	352–500	445	378–523	585	371–921	854	697–1044	515	484–546	791	668–936
Primary	360	278–464	347	273–439	720	332–1548	792	617–1014	394	359–431	542	391–751
Secondary	148	115–190	232	189–284	237	130–427	463	393–545	203	188–217	294	220–392
Higher	46	25–81	220	108–443	482	161–1430	450	316–639	96	79–116	221	100–484
Wealth quintile												
Poorest	394	321–481	384	333–443	244	117–506	661	537–812	547	510–584	716	599–855
Poorer	233	182–296	316	249–401	460	246–856	554	459–668	372	344–402	543	425–691
Middle	267	210–339	247	182–335	674	404–1121	623	494–783	252	228–278	278	202–381
Richer	197	145–266	254	137–468	690	311–1521	552	420–723	197	177–219	241	121–474
Richest	116	72–185	65	28–149	30	6–141	619	415–919	149	130–169	354	157–791
Place of residence												
Urban	207	158–268	273	196–381	571	340–958	751	609–925	215	196–236	452	285–715
Rural	234	205–266	336	298–378	366	239–558	572	503–649	325	310–338	508	446–578
Household size												
1–3 Members	297	241–364	529	430–649	918	599–1402	752	615–917	349	322–377	676	510–894
4–5 Members	194	155–241	296	244–359	228	115–448	503	423–599	258	241–275	442	358–544
6 & above Members	206	166–254	256	213–306	278	126–608	623	513–756	282	263–300	456	372–558
Persons sleeping per room												
< 3 person	245	214–280	335	293–381	480	322–713	624	531–733	313	299–327	526	453–610
3–4 person	122	91–163	299	240–372	338	185–615	567	489–656	219	199–239	418	314–556
> 5 person	153	72–323	420	229–764	157	38–648	756	516–1107	190	140–255	176	49–620
Type of house												
Natural	298	245–361	373	323–429	354	242–515	590	499–697	427	396–459	619	516–741
Rudimentary	281	217–362	341	257–451	568	308–1040	840	691–1020	362	338–387	448	341–586
Finished	191	159–229	246	192–314	447	223–891	474	383–586	214	200–228	419	324–541
Separate kitchen												
Yes	170	141–205	258	216–307	429	268–686	592	509–687	225	211–238	359	289–444
No	322	254–406	387	318–470	217	126–370	697	537–903	406	378–435	568	448–720
Type of fuel used for cooking												
Improved	208	174–246	242	182–321	635	431–933	691	593–804	221	207–235	323	237–438
Unimproved	261	222–305	359	317–405	242	129–450	557	477–649	381	362–401	621	538–715
Potability of water												
Yes	198	163–240	277	239–320	330	204–530	642	571–721	226	209–243	353	285–437
No	254	216–298	398	335–470	639	410–993	480	355–647	325	310–341	592	502–697
Shared toilet												
No	199	170–232	267	228–311	393	268–574	601	538–669	244	232–256	390	323–470
Yes	275	164–457	447	262–759	1038	471–2269	879	646–1195	331	288–380	693	385–1241
Smoking inside the house												
Never	180	151–213	295	247–350	268	130–549	623	511–757	241	227–255	390	319–475
Yes	303	252–362	360	310–416	563	395–801	602	527–685	347	328–366	601	504–716
Diabetes												
No	206	177–240	312	274–354	443	307–637	554	492–622	282	268–295	500	433–577
Yes	373	290–479	469	349–628	423	185–958	941	670–1319	406	366–448	598	371–962
Hypertension												
No	224	194–258	319	279–364	344	229–516	577	505–657	295	280–309	525	449–611
Yes	240	179–320	392	311–493	671	353–1273	645	529–786	312	286–339	465	345–627
Consumption of alcohol												
No	195	170–222	262	226–303	338	225–504	611	539–692	259	247–270	394	339–457
Yes	499	367–677	580	486–691	844	475–1493	598	476–750	571	520–626	1008	777–1306
Consumption of tobacco												
No	173	146–203	220	185–262	240	139–411	524	449–610	218	206–229	340	283–406
Yes	414	342–500	517	445–598	826	545–1247	724	618–845	528	496–560	847	699–1026
Overall	224	197–253	330	294–368	422	303–585	608	545–678	287	275–298	498	436–568

*NFHS* National Family Health Survey, *ST* Schedule Tribe, *CI* Confidence Interval

In non-scheduled districts with > 60% STs, the point prevalence of TB per 100,000 persons was consistently higher among tribals across key characteristics compared to non-tribals (Table 3). Similar to scheduled area districts, TB prevalence increased with age in both groups, with STs showing higher prevalence in every age category, notably 1197 (95% *CI* 984–1452) in the 60 + group compared to 840 (95% *CI* 406–1730) among non- STs. Illiterates had the highest prevalence, with tribals at 854 (95% *CI* 697–1044) and non-tribals at 585 (95% *CI* 371–921). Among wealth quintiles, the poorest tribal group (661, 95% *CI* 537–812) had a higher prevalence than the poorest non-tribals (244, 95% *CI* 117–506). Households using unimproved fuel showed higher prevalence among tribals (557, 95% *CI* 477–649) than non-tribals (242, 95% *CI* 129–450). In non-scheduled districts with < 60% STs population, TB point prevalence per 100,000 persons was consistently higher among tribals across key categories. The 60 + age group had a prevalence of 835 (95% *CI* 637–1094) among tribals, compared to 579 (95% *CI* 540–619) in non-tribals, while tribal males had a higher prevalence (649, 95% *CI* 549–766) than non-tribal males (371, 95% *CI* 353–389). Illiterate STs exhibited a striking prevalence of 791 (95% *CI* 668–936) against 515 (95% *CI* 484–546) in non-STs. The poorest tribal groups had a higher prevalence (716, 95% *CI* 599–855) than their non-tribal counterparts (547, 95% *CI* 510–584). Alcohol consumption was associated with a significantly higher prevalence among STs (1008, 95% *CI* 777–1306) compared to non-STs (571, 95% *CI* 520–626).

### Determinants of TB

STs had significantly higher odds of having TB compared to non-STs across all categories: *aOR* = 1.57 (95% *CI* 1.37–1.79; *P* < 0.001) in Scheduled Areas, *aOR* = 1.09 (95% *CI* 0.87–1.37; *P* = 0.448) in districts with more than 60% STs, *aOR* = 1.20 (95% *CI* 1.08–1.33;; *P* < 0.001) in districts with less than 60% STs, and *aOR* = 1.47 (95% *CI* 1.38–1.56; *P* < 0.001) for the total study population (Table 4). We found that the majority of the selected independent variables, particularly those related to socioeconomic status, were strongly associated with TB. However, Scheduled Areas consistently exhibited lower estimated odds compared to their non-Scheduled Area counterparts for the same categories of independent variables. This suggests that while the associations remain statistically significant, the strength of these associations tends to be lower in Scheduled Areas. For instance, compared to individuals aged 15–29 years, those aged 60 years and above had significantly higher odds of TB across all district categories. [(Scheduled Area- a*OR* = 2.82, 95% *CI* 2.27–3.51; *P* < 0.001), (districts marked with > 60% STs- a*OR* = 3.37; 95% *CI* 2.61–4.34; *P* < 0.001) and (districts marked with < 60% STs- a*OR* = 3.60; 95% *CI* 3.23–4.02; *P* < 0.001)]. Similarly, in the case of education, individuals with secondary education had higher odds of TB compared to those who were illiterate. The estimated odds for secondary education were a*OR* = 1.75 (95% *CI* 1.27–2.41; *P* < 0.001) in Scheduled Areas, whereas the corresponding odds were notably higher at a*OR* = 2.26 (95% *CI* 1.91–2.68; *P* < 0.001) in districts with less than 60% STs, reflecting the same pattern of lower magnitude in Scheduled Areas. The odds for wealth status revealed the clear and consistent gradient in TB odds across wealth quintiles, particularly in Scheduled Areas and districts with less than 60% STs population. Compared to the richest group, the poorest individuals had significantly higher odds of TB (a*OR* = 2.02, 95% *CI* 1.34 − 3.04; *P* < 0.001) in Scheduled Areas and—in < 60% STs districts (a*OR* = 2.55; 95% *CI* 2.15–3.02; *P* < 0.001), while the association was weaker and not statistically significant in > 60% STs districts. A similar pattern is observed across the poorer, middle, and richer groups, with higher and significant odds in Scheduled and < 60% STs districts, but not significant associations in > 60% STs districts.

**Table 4:** Adjusted odds ratio estimated for the determinants of medically treated tuberculosis in India, NFHS-5 (2019–2021)

	Scheduled area districts			Districts with > 60% sts			Districts with < 60% sts			Total		
	a*OR*	95% *CI*	*P*-value	a*OR*	95% *CI*	*P*-value	a*OR*	95% *CI*	*P*-value	a*OR*	95% *CI*	*P*-value
Explanatory variables												
Ethnicity												
Non–STs (ref.)												
STs	1.57	1.37–1.79	< 0.001	1.09	0.87–1.37	0.448	1.20	1.08–1.33	< 0.001	1.47	1.38–1.56	< 0.001
Age												
15–29 (ref.)												
30–44	1.70	1.4–2.05	< 0.001	1.81	1.45–2.26	< 0.001	1.55	1.4–1.72	< 0.001	1.64	1.5–1.78	< 0.001
45–59	2.44	2–2.98	< 0.001	2.43	1.93–3.06	< 0.001	2.59	2.33–2.88	< 0.001	2.56	2.35–2.79	< 0.001
60 +	2.82	2.27–3.51	< 0.001	3.37	2.61–4.34	< 0.001	3.60	3.23–4.02	< 0.001	3.46	3.16–3.79	< 0.001
Sex												
Female (ref.)												
Male	1.77	1.54–2.04	< 0.001	1.61	1.39–1.87	< 0.001	1.93	1.79–2.08	< 0.001	1.85	1.74–1.97	< 0.001
Education												
Illiterate (ref.)												
Primary	1.19	0.88–1.6	0.263	0.98	0.74–1.29	0.880	1.58	1.35–1.85	< 0.001	1.38	1.22–1.56	< 0.001
Secondary	1.75	1.27–2.41	< 0.001	1.35	0.98–1.85	0.064	2.26	1.91–2.68	< 0.001	1.95	1.7–2.23	< 0.001
Higher	1.58	1.14–2.18	0.006	1.76	1.28–2.41	< 0.001	2.56	2.16–3.02	< 0.001	2.12	1.85–2.42	< 0.001
Wealth quintile												
Richest (ref.)												
Richer	1.51	1.08–2.11	0.015	1.02	0.71–1.47	0.906	1.16	1.02–1.32	0.028	1.27	1.13–1.42	< 0.001
Middle	2.01	1.44–2.8	< 0.001	1.24	0.86–1.79	0.254	1.40	1.23–1.6	< 0.001	1.62	1.45–1.82	< 0.001
Poorer	1.88	1.31–2.71	< 0.001	1.24	0.82–1.87	0.304	1.87	1.62–2.16	< 0.001	1.96	1.73–2.23	< 0.001
Poorest	2.02	1.34–3.04	< 0.001	1.46	0.93–2.3	0.103	2.55	2.15–3.02	< 0.001	2.42	2.09–2.8	< 0.001
Place of residence												
Rural (ref.)												
Urban	1.36	1.13–1.64	0.001	1.31	1.08–1.58	0.005	1.19	1.09–1.3	< 0.001	1.27	1.18–1.37	< 0.001
Household size												
1–3 members (ref.)												
4–5 members	0.88	0.76–1.02	0.095	0.89	0.75–1.05	0.156	1.05	0.96–1.13	0.284	0.97	0.91–1.03	0.305
6 & above members	0.82	0.7–0.96	0.016	1.06	0.88–1.27	0.555	1.02	0.94–1.11	0.636	0.94	0.88–1.01	0.078
Persons sleeping per room												
< 3 person (ref.)												
3–4 person	1.03	0.88–1.2	0.731	1.02	0.88–1.19	0.767	0.97	0.9–1.06	0.530	1.04	0.98–1.11	0.187
> 5 person	1.20	0.83–1.75	0.336	1.19	0.88–1.62	0.264	0.96	0.78–1.19	0.714	1.15	0.99–1.35	0.075
Type of house												
Finished (ref.)												
Rudimentary	1.10	0.92–1.31	0.301	1.71	1.39–2.11	< 0.001	1.14	1.05–1.24	0.002	1.25	1.16–1.34	< 0.001
Natural	0.96	0.81–1.15	0.683	1.32	1.06–1.64	0.015	1.05	0.96–1.16	0.295	1.08	1–1.17	0.045
Separate kitchen												
Yes (ref.)												
No	1.22	1.06–1.42	0.008	0.99	0.84–1.18	0.952	1.14	1.05–1.23	0.001	1.16	1.09–1.24	< 0.001
Type of fuel used for cooking												
Improved (ref.)												
Unimproved	0.91	0.77–1.08	0.299	0.66	0.54–0.8	< 0.001	0.90	0.83–0.98	0.012	0.84	0.78–0.9	< 0.001
Potability of water												
Yes (ref.)												
No	1.00	0.89–1.13	0.993	0.87	0.72–1.05	0.138	1.05	0.98–1.13	0.142	0.98	0.93–1.04	0.582
Shared toilet												
No (ref.)												
Yes	1.24	0.97–1.57	0.082	1.75	1.41–2.19	< 0.001	1.16	1.04–1.29	0.009	1.24	1.13–1.36	< 0.001
Smoking inside the house												
Never (ref.)												
Yes	1.32	1.16–1.5	< 0.001	1.03	0.88–1.21	0.692	1.10	1.03–1.18	0.003	1.17	1.11–1.24	< 0.001
Diabetes												
No (ref.)												
Yes	1.33	1.13–1.57	0.001	0.91	0.73–1.12	0.368	1.13	1.04–1.23	0.006	1.13	1.05–1.22	0.001
Hypertension												
No (ref.)												
Yes	0.80	0.69–0.93	0.004	0.85	0.73–1.00	0.057	0.75	0.7–0.82	< 0.001	0.78	0.73–0.83	< 0.001
Consumption of alcohol												
No (ref.)												
Yes	1.05	0.9–1.22	0.511	0.94	0.79–1.11	0.463	1.17	1.08–1.28	< 0.001	1.08	1.01–1.15	0.030
Consumption of tobacco												
No (ref.)												
Yes	1.38	1.2–1.59	< 0.001	1.10	0.93–1.29	0.260	1.10	1.02–1.19	0.016	1.16	1.09–1.23	< 0.001

## Discussion

Using a large, nationally representative dataset, we aimed to generate evidence on the persistent inequities in TB burden faced by India’s tribal communities. Efforts to eliminate tuberculosis through an equity-focused approach are often constrained by the lack of reliable, disaggregated data to guide the design, delivery, and evaluation of interventions for marginalised populations [22]. Our study carries this agenda by quantifying disparities at both national and sub-national levels and by applying a novel district classification framework to explore structural and geographic dimensions of TB inequity. One of the key contributions of this research is exploration of the role of constitutionally protected tribal regions known as Scheduled Areas in the epidemiology of TB.

We observed that among districts designated as Scheduled Areas there had a lower overall TB prevalence among tribals compared to non-Scheduled districts with high tribal populations. This suggests that the administrative and governance structures in Scheduled Areas context which may include better allocation of resources, targeted interventions or empowered local institutions could provide certain community health benefits. Scheduled Areas benefit from special administrative mechanisms such as Tribes Advisory Councils and empowered panchayats under the Panchayats (Extension to Scheduled Areas) PESA Act, 1996 in Fifth Schedule states and Autonomous District Councils in Sixth Schedule areas, which are intended to ensure that tribal needs are addressed within local self-governance frameworks [19, 23]. Through self-governance, *Panchayats* have constitutional mandates for improving healthcare service delivery in the community through Health Sub Centres, Health & Wellness Centres /Ayushman Arogya Mandirs, monitoring through sub-committees called Village Health Sanitation and Nutrition Committees and Jan Arogya Samitis and health and community mobilization [23].

Conversely, in non-Scheduled districts with a high STs concentration, we documented the worst TB outcomes with both tribals and non-tribal populations experiencing the highest prevalence rates. The high TB burden there could be attributed to minimal access to healthcare, persistent poverty, and negligible influence of tribal voices in local decision-making [17, 24]. Tribals in these districts might also experience social marginalization since they lack autonomous council representation like protected districts under Schedule Areas. Our data showed that even non-tribal residents of these districts had high TB prevalence, indicating that the entire district likely suffers from poor health infrastructure and risk factors which disproportionately affect tribals even more. This finding suggests that targeting resources to high-tribal-concentration districts -even if they are not officially Scheduled Areas- should be a priority for TB elimination efforts. Currently, government initiatives like the Tribal TB Initiative [15] focus on identified tribal pockets; but our results can help refine those targets by identifying high-risk districts that might have been overlooked due to lack of Schedule V or VI status. Further, in non-Scheduled districts with low tribal concentrations, the difference in TB prevalence between STs and non-STs is stark, indicating higher marginalization of tribals in absence of any constitutional or other local socio-political support structures.

Our bivariate analysis across key socioeconomic, demographic, and proximate characteristics reinforces previous findings on the social determinants of tuberculosis. TB prevalence was notably higher among males compared to females [25, 26], and this gender disparity was even more pronounced among tribal populations [10, 27]. Age also exhibited a clear gradient, with prevalence increasing steadily across age groups [25], again with a stronger effect observed among tribals-consistent with patterns reported in earlier studies [25]. Rural residents had a higher prevalence of TB than their urban counterparts, highlighting persistent inequities in access to healthcare services in rural India [25, 27]. Household environmental conditions were also strongly associated with TB burden. Individuals living in natural or kuccha houses, lacking a separate kitchen, using unimproved cooking fuels, relying on non-potable water, or sharing toilet facilities exhibited higher TB prevalence [28]. In addition, proximate health determinants such as diabetes and hypertension, along with behavioural risk factors including alcohol and tobacco use, were significantly associated with elevated TB risk, aligning with existing literature on comorbidity and lifestyle influences [29]. Across nearly all dimensions, tribal populations experienced disproportionately worse outcomes compared to their non-tribal counterparts, underscoring the compounded disadvantage they face in both social and health domains [10, 15–17, 30–32]. Further, our stratified analysis indicates that the highest absolute prevalence is observed among tribals in non-Scheduled districts with more than 60% STs population, at 608/100,000, compared to 422/100,000 among non-STs, accounted for 44% of relative difference. In non-Scheduled districts with less than 60% STs population, tribal prevalence remains high at 498 per 100,000, but the disparity with non-STs (287 per 100,000) is even more pronounced, reflecting the highest relative difference of 73.5%. In contrast, constitutionally recognised Scheduled Areas under the Fifth and Sixth Schedules show comparatively lower TB prevalence among tribals (330 per 100,000) and a more moderate gap with non-STs (224 per 100,000), with a relative difference of 47%. This result reflects the fact that the TB burden among tribal populations is driven by both absolute deprivation and relative disadvantage, with significant variation across district types. A similar finding was suggested in a study that vulnerable populations are those with a high risk for tuberculosis disease and whose disadvantaged or marginalised socioeconomic position limits their access to the health system [33].

Data from this study also indicate that tribal identity itself appears to be a salient determinant of TB risk. Even after adjusting for various socioeconomic, demographic and household environmental factors in our regression models, tribals had significantly higher odds of TB compared to non-tribals. This suggests that there are unmeasured structural and systemic factors inherent to tribal communities that contribute to TB vulnerability [34, 35]. Possible factors include systemic inequalities e.g. tribals facing discrimination or lower quality of care in health facilities, nutritional disparities, and historical neglect leading to lower awareness and health-seeking behavior for TB [9, 10, 15, 36, 37]. Another contributor could be differences in exposure- many tribals live and work in high-risk environments such as mines, quarries, or forests, which might increase exposure to TB related conditions [38]. The finding that even the wealthier or more educated tribals sometimes had higher TB odds than less educated non-tribals contrary to typical gradients highlights how being a tribal person might entail unique risks that transcend standard socio-economic indicators [31] which is not merely a byproduct of poverty or rural living, but an additional layer of vulnerability [39, 40]. Studies have shown that the stress generated by social inequality leads to persistent inflammation which leads to poor health outcomes [41]. Furthermore, some literature highlights that chronic stress arising from social exclusion and inequality can trigger persistent systemic inflammation, thereby increasing susceptibility to a range of diseases, including infections such as TB [41, 42].

Disaggregating by wealth quintiles, logistic regression analysis reveals distinct patterns in the relationship between economic status and TB risk. In Scheduled Areas, there is a statistically significant gradient, with the poorest tribals showing twice the odds of TB compared to the richest (a*OR* = 2.02, 95% *CI* 1.34–3.04, *P* < 0.001), and all other lower wealth groups also exhibiting significantly elevated odds. Non-Scheduled districts with less than 60% STs population, however, display a steeper and statistically robust wealth gradient. The poorest STs have 2.55 times the odds of TB compared to the richest (95% *CI* 2.15–3.02, *P* < 0.001), with consistently elevated and significant odds observed across all lower wealth quintiles. While direct comparisons of adjusted odds ratios between these two district types are limited by differences in model specification and base population, the pattern of stronger associations and statistical significance in non-Scheduled districts suggests a more pronounced impact of socioeconomic disadvantage where institutional protections are absent. These findings indicate that economic advancement alone does not mitigate health disparities, particularly in contexts characterised by structural exclusion [43]. In Scheduled Areas, legal safeguards, cohesive governance structures, and targeted public health interventions may buffer the health consequences of poverty. In contrast, tribal populations in non-Scheduled districts, especially those who are politically and demographically marginal, face compounded vulnerabilities arising from the convergence of economic deprivation, social exclusion, and institutional neglect [44]. Addressing these disparities demands not only poverty alleviation, but also structural reforms, including the extension of constitutional protections and programmatic prioritisation for underserved tribal dominated geographies beyond the Scheduled Area framework.

The Global TB Report 2024 noted an upsurge in TB cases worldwide in 2023, the highest since 1995, attributable in part to pandemic-related setbacks [7]. India’s tribal areas, already underserved, likely experienced even greater disruptions during COVID-19, potentially slowing progress in TB detection and treatment [45]. Our trend analysis of NFHS data showed that improvements in TB prevalence from 2016 to 2021 were uneven—slower among tribals and even reversed in some states. For example, we found that in states such as Assam, Gujarat, Rajasthan, Karnataka, West Bengal and across much of the Northeast, TB prevalence among tribals actually increased in recent years. These regions coincide with either high tribal populations or difficult-to-reach terrain (e.g., Northeast India’s hills and forests), and some also have high humidity and rainfall. Interestingly, emerging research suggests climatic factors like high humidity can facilitate TB transmission by prolonging droplet nuclei suspension and viability of Mycobacterium tuberculosis [46]. Regions like the Northeast and parts of coastal/western India are humid and saw high or rising TB rates, consistent with studies linking climate variables to TB incidence [46, 47]. While climate is not a modifiable factor in the short term, recognizing such geographic patterns can help in tailoring region-specific TB control strategies. For instance, interventions in humid regions could emphasize improved housing ventilation to reduce indoor transmission risks. Furthermore, the success of Madhya Pradesh which recorded one of the largest declines in tribal TB prevalence could offer lessons. The state’s approach included close collaboration with the existing health system and strong integration with the TB control programme has significantly contributed to the reduction of TB, even in predominantly tribal regions [48].

Our findings suggest that the effective implementation of public health programs, including TB control initiatives, can be more successful when they are inclusive of local cultural contexts and tailored to reach in remote villages. Indeed, our results hint that tribals living in Scheduled area districts, still having higher TB rates compared to non-tribals, they fare better than their counterparts in non-Scheduled high-tribal districts where such constitutional protections are absent. This “protective” effect of Scheduled Area status is a novel insight reinforcing the notion that administrative and legal empowerment of marginalized groups can translate into better health outcomes, at least at the aggregate level. For example, The Sixth Schedule of the Constitution of India empowers local governments to make decisions related to health and family welfare including public health and sanitation, hospitals and dispensaries, public health engineering, and related services [20]. The PESA Act, [49, 50] was specifically enacted to empower tribal local self-governance in Fifth Schedule areas, and evidence suggests it has led to improvements in certain indicators related to health, income and education [51]. However, our findings also show that within Scheduled Areas, tribals still have significantly higher TB prevalence than non-tribals indicating that the protections and autonomy granted in these areas have not fully eliminated health inequalities. The persistence of this gap is likely attributed to ongoing socio-economic disparities and potentially under-resourced health systems in these regions [24, 52]. This also highlights the role of social deter related to power and participation which, through multitudinous pathways, is influencing prevalence of TB in the community.

The findings of this study indicate important policy implications for India’s TB elimination efforts, particularly in the context of tribal communities. To achieve the End TB Strategy goals and SDG target 3.3 (ending the TB epidemic by 2030), India must prioritize tribal communities in its TB elimination roadmap. This entails multiple actions such as enhancing TB surveillance and case finding in tribal areas, ensuring that every tribal-dominated district has well-equipped TB diagnostic centres and treatment through directly observed therapy. Mobile TB clinics and camps can be deployed in remote villages to bridge healthcare access gap. Given the high vulnerability of older tribals, age-targeted interventions like periodic screenings in elder tribal populations could be beneficial. Our data confirm that older age amplifies TB risk in tribals, partly due to comorbidities and cumulative exposure. This might be addressed by integrated care for diseases of the elderly in primary health centres in the tribal areas.

Another key strategy is strengthening community engagement and ownership of TB programs. Community health workers and traditional healers should be trained to recognize TB symptoms and refer suspected cases, can improve early detection and reduce diagnostic delays. The success of community-led initiatives in some Scheduled Areas (like self-help groups and peer support groups for TB awareness) can be expanded. We noted that self-governance alone (as via PESA) is not sufficient to curb TB; it must be accompanied by investment in healthcare infrastructure. For example, more TB units and microscopy centres in tribal blocks, sufficient staffing of nurses and outreach workers who speak local tribal languages, and use of technology (like telemedicine or digital adherence tools) designed for low-literacy settings. While government programs like Ni-Kshay provide nutritional support for TB patients, integrating culturally appropriate dietary interventions into TB care could enhance effectiveness in the tribal areas [53]. Moreover, addressing the broader social determinants of TB such as poverty, inadequate housing and poor sanitation must be central to the strategy. Such efforts align with the WHO End TB Strategy’s call to tackle the root causes of TB. Our study vividly demonstrates that without improving living conditions and targeted efforts to reduce systemic inequities, medical interventions alone may not be sufficient to eliminate TB in tribal communities.

This study has certain limitations. First, reliance on self-reported TB status in NFHS-5 may lead to underreporting or misclassification, particularly due to stigma or undiagnosed cases, resulting in an underestimation of the actual TB burden. Second, although we adjusted for multiple covariates, residual confounding remains possible-particularly from unmeasured factors like malnutrition, HIV status, or genetic susceptibility to TB. Third, the cross-sectional nature of NFHS-5 limits causal inference; observed associations may reflect reverse causality. Fourth, our classification of Scheduled Areas at the district level was an approximation and may mask within-district variation. Fifth, as we focused on prevalence, we could not assess TB incidence or treatment outcomes in the study. Lastly, although no major distortions were observed, the potential indirect impact of the COVID-19 pandemic on TB diagnosis and reporting during the survey period cannot be entirely ruled out. Despite these constraints, the study uses a nationally representative dataset and a novel district-level framework to generate valuable, policy-relevant insights. The consistent disadvantage observed among STs across analyses reinforces the validity of our findings. Future research should incorporate microbiologically confirmed TB case data and explore longitudinal and intervention-based approaches to address the disparities highlighted in this study.

## Conclusions

This study demonstrates that STs populations in India have significantly higher TB prevalence than non-STs across various district categories, driven by a confluence of social, household, environmental, and structural factors. We also found that districts with constitutional protections for STs (Scheduled Areas) tend to have better TB outcomes than other tribal-dominated areas, suggesting that governance mechanisms and focused resources can make a difference [23]. However, substantial inequities remain, highlighting that existing strategies need to be more responsive to the unique vulnerabilities of tribal populations. Addressing the burden of TB in the tribal communities is not only a public health necessity but also a matter of social equity and human rights. By focusing on those ‘left behind’, herein this case the tribal communities in both Scheduled and non-Scheduled areas, India can accelerate its fight against TB. Success from these interventions would contribute directly to achieving SDG 3.3, which calls for ending the TB epidemic, and SDG 10, which emphasizes reduction of inequalities. Ultimately, eliminating TB in India will require reducing the socio-economic and structural inequities that have perpetuated the disease in its most marginalized communities. Our findings serve as evidence and a call to action to ensure that tribal communities need to be placed at the forefront of TB control initiatives in the years to come, so that the burden of TB no longer disproportionately affects those already facing socio-economic hardships.

## Supplementary Information

Supplementary material 1.

## Data Availability

The data utilized in this study are publicly available from the DHS upon request and filing the registration. DHS data are available on: https://dhsprogram.com/data/available-datasets.cfm.

## Abbreviations

ADCs: Autonomous Districts Councils
a*OR*: Adjusted odds ratio
*CI*: Confidence interval
DHS: Demographic and Health Survey
DOT: Directly observed therapy
HWCs: Health and Wellness Centres
JAS: Jan Arogya Samiti
NFHS: National Family Health Survey
*OR*: Odds ratio
PESA: Panchayats (Extension to Scheduled Areas) Act
SDG: Sustainable development goals
STs: Schedule Tribes
TACs: Tribes Advisory Councils
TB: Tuberculosis
UT: Union Territories
VHSNCs: Village Health Sanitation & Nutrition Committee
WHO: World Health Organization
