# Supplementary material for: Burden on the burdened: tuberculosis among Scheduled Tribes and non-Scheduled Tribes in constitutionally protected Scheduled and non-Scheduled areas of India
Source: Infect Dis Poverty. 2025 Nov 3;14:111. doi: 10.1186/s40249-025-01375-9 (PMC12581512; doi:10.1186/s40249-025-01375-9)
Supplement: Supplementary file 1 — Supplementary material 1. [file 40249_2025_1375_MOESM1_ESM.docx]

**Supplementary Table 1: Detailed description of the selected variables used in the study, NFHS-5 (2019­­–2021)**

| **Variables included in the study** | **Description** |
| --- | --- |
| **Outcome variable** |  |
| Tuberculosis (TB) | Has any usual member of your household, including yourself, received medical treatment for tuberculosis? Categorised as 'Yes = 1' or 'No = 0'. |
| **Exposure variable** |  |
| Ethnic group | The NFHS collects information on individuals' social group affiliation by asking the question: 'Is this person a member of a Scheduled Caste (SCs), a Scheduled Tribe (STs), an Other Backward Class (OBC), or none of these?' For analysis, the responses were categorised as ST and Non-STs. |
| Categorization of districts | Based on the concentration of the tribal population, districts were classified into three groups: (i) districts designated under the Scheduled Areas (i.e., those falling under Schedule 5 or Schedule 6); (ii) districts with more than 60% tribal population; and (ii) districts with less than 60% tribal population. |
| **Independent Variables** |  |
| Age | The self-reported age of individuals was recorded and categorised into four groups: 15–29 years, 30–44 years, 45–59 years, and 60 years and above |
| Sex | Individual's sex was categorized into male and female |
| Education | Based on the level of educational attainment, individuals were classified into four categories: no education, primary education, secondary education, and higher education. |
| Wealth quintile | Information on participants’ ownership of consumer goods and other household characteristics was used to compute household wealth. The resulting wealth index was then categorised into five groups: poorest, poorer, middle, richer, and richest. |
| Place of residence | Participant's current place of residence was categorized into urban and rural. |
| Household size | The household size was categorized into 1-3 members, 4-5 members, and more than 5 members. |
| Number of persons sleeping per room | The number of sleeping rooms in the house was categorized as less than 3-persons, 3-4 persons and more than 5 persons per room. |
| Type of house | Housing type was classified as natural, rudimentary, or finished, based on the materials used for the roof, walls, and floor as reported by respondents. |
| Separate kitchen | The separate kitchen in the household was categorized into “Yes” or “No”. |
| Type of fuel used for cooking | The primary type of cooking fuel used in the household was recorded and categorised as improved (e.g., electricity, LPG/natural gas, biogas) or non-improved (e.g., kerosene, coal/lignite, charcoal, wood, straw/shrubs/grass, agricultural crop waste, and dung cakes). |
| Potability of water | Households were asked about the methods they usually use to make water safer for drinking, such as boiling, using alum, adding bleach or chlorine tablets, straining through a cloth, using a water filter, electronic purifier, solar disinfection, etc. Based on these responses, drinking water potability was categorised as 'Yes' or 'No'. |
| Shared toilet | Respondents were asked whether their household shares the toilet facility with other households. Responses were categorised as 'Yes' or 'No'. |
| Smoking inside the house | Respondents were asked whether they or anyone else had smoked inside the home in the past 30 days. Responses were recorded as 'Yes' or 'No'. |
| Diabetes | Biomarker data on diabetes among men and women aged 15 years and above were collected in NFHS-5. According to standard criteria, individuals were classified as diabetic if their random blood glucose level exceeded 140 mg/dl or if they were taking medication to lower blood glucose levels. |
| Hypertension | Biomarker data on hypertension among men and women aged 15 years and above were collected in NFHS-5. Individuals were classified as hypertensive if their diastolic blood pressure (DBP) was ≥90 mmHg or systolic blood pressure (SBP) was ≥140 mmHg at the time of the survey, or if they were on medication to control blood pressure. |
| Consumption of alcohol | Respondents were asked whether they currently consume alcohol. Responses were recorded as 'Yes' or 'No'. |
| Consumption of tobacco | Respondents were asked whether they currently smoke or use tobacco in any form. Responses were recorded as 'Yes' or 'No'. |

Note: NFHS National Family Health Survey, ST Schedule Tribe

**Supplementary Table 2: Sample description of the study population aged 15 and above by STs, non-STs and total, in India, NFHS-5 (2019­­–2021)**

|  | Non-STs |  | STs |  | Total |  |
| --- | --- | --- | --- | --- | --- | --- |
| Characteristics | % (weighted) | N (Unweighted) | % (weighted) | N (Unweighted) | % (weighted) | N (Unweighted) |
| **Age** |  |  |  |  |  |  |
| 15-29 | 35.3 | 6,02,469 | 37.7 | 1,37,521 | 35.5 | 7,39,990 |
| 30-44 | 27.4 | 4,66,368 | 28.0 | 1,07,603 | 27.4 | 5,73,971 |
| 45-59 | 21.2 | 3,59,427 | 20.5 | 81,324 | 21.1 | 4,40,751 |
| 60+ | 16.1 | 2,72,925 | 13.8 | 50,287 | 15.9 | 3,23,212 |
| **Sex** |  |  |  |  |  |  |
| Female | 51.4 | 8,71,052 | 51.6 | 1,93,251 | 51.4 | 10,64,303 |
| Male | 48.7 | 8,30,137 | 48.4 | 1,83,484 | 48.6 | 10,13,621 |
| **Education** |  |  |  |  |  |  |
| Illiterate | 23.6 | 4,20,471 | 35.9 | 1,16,402 | 24.7 | 5,36,873 |
| Primary | 13.6 | 2,25,220 | 14.8 | 56,800 | 13.7 | 2,82,020 |
| Secondary | 47.3 | 8,12,566 | 41.7 | 1,72,085 | 46.8 | 9,84,651 |
| Higher | 15.5 | 2,42,932 | 7.7 | 31,448 | 14.8 | 2,74,380 |
| **Wealth Quintile** |  |  |  |  |  |  |
| Poorest | 15.5 | 2,73,969 | 43.9 | 1,48,768 | 18.1 | 4,22,737 |
| Poorer | 18.9 | 3,45,520 | 24.8 | 1,03,037 | 19.4 | 4,48,557 |
| Middle | 20.7 | 3,62,237 | 15.8 | 66,044 | 20.3 | 4,28,281 |
| Richer | 21.9 | 3,59,998 | 9.5 | 40,560 | 20.7 | 4,00,558 |
| Richest | 23.1 | 3,59,465 | 6.1 | 18,326 | 21.5 | 3,77,791 |
| **Place of Residence** | |  |  |  |  |  |
| Urban | 35.0 | 4,74,392 | 14.1 | 50,802 | 33.1 | 5,25,194 |
| Rural | 65.0 | 12,26,797 | 85.9 | 3,25,933 | 66.9 | 15,52,730 |
| **Household Size** |  |  |  |  |  |  |
| 1-3 Members | 21.9 | 3,52,901 | 21.4 | 90,888 | 21.8 | 4,43,789 |
| 4-5 Members | 40.1 | 6,85,690 | 41.2 | 1,57,760 | 40.2 | 8,43,450 |
| 6 & above Members | 38.0 | 6,62,598 | 37.4 | 1,28,087 | 38.0 | 7,90,685 |
| **Person Sleeping per Room** | | |  |  |  |  |
| <3 person | 74.1 | 12,09,498 | 77.2 | 2,43,540 | 74.4 | 14,53,038 |
| 3-4 person | 23.0 | 4,36,530 | 20.6 | 1,18,628 | 22.8 | 5,55,158 |
| >5 person | 2.9 | 55,161 | 2.2 | 14,567 | 2.8 | 69,728 |
| **Type of House** |  |  |  |  |  |  |
| Natural | 16.2 | 3,27,049 | 45.0 | 1,80,990 | 18.9 | 5,08,039 |
| Rudimentary | 24.5 | 4,18,199 | 20.6 | 77,663 | 24.2 | 4,95,862 |
| Finished | 59.2 | 9,55,941 | 34.4 | 1,18,082 | 57.0 | 10,74,023 |
| **Separate Kitchen** |  |  |  |  |  |  |
| Yes | 59.2 | 9,92,817 | 49.2 | 1,89,665 | 58.2 | 11,82,482 |
| No | 22.1 | 3,60,785 | 26.0 | 89,319 | 22.5 | 4,50,104 |
| Missing | 18.7 | 3,47,587 | 24.9 | 97,751 | 19.3 | 4,45,338 |
| **Type of Fuel used for cooking** | |  |  |  |  |  |
| Improved | 60.7 | 9,64,745 | 32.7 | 1,29,079 | 58.2 | 10,93,824 |
| Unimproved | 39.3 | 7,36,444 | 67.3 | 2,47,656 | 41.8 | 9,84,100 |
| **Potability of water** | |  |  |  |  |  |
| Yes | 41.3 | 7,28,086 | 50.2 | 2,33,813 | 42.2 | 9,61,899 |
| No | 58.7 | 9,73,103 | 49.8 | 1,42,922 | 57.9 | 11,16,025 |
| **Shared Toilet** |  |  |  |  |  |  |
| No | 75.7 | 13,04,578 | 61.3 | 2,76,950 | 74.4 | 15,81,528 |
| Yes | 8.0 | 1,30,125 | 5.2 | 19,654 | 7.8 | 1,49,779 |
| Missing | 16.2 | 2,66,486 | 33.4 | 80,131 | 17.8 | 3,46,617 |
| **Smoking inside the House** | |  |  |  |  |  |
| Never | 58.1 | 9,28,761 | 46.5 | 1,44,453 | 57.0 | 10,73,214 |
| Yes | 41.9 | 7,72,416 | 53.5 | 2,32,282 | 43.0 | 10,04,698 |
| Diabetes |  |  |  |  |  |  |
| No | 73.7 | 12,84,274 | 79.5 | 3,08,651 | 74.3 | 15,92,925 |
| Yes | 11.7 | 1,84,600 | 9.0 | 34,654 | 11.4 | 2,19,254 |
| Missing | 14.6 | 2,32,315 | 11.5 | 33,430 | 14.3 | 2,65,745 |
| **Hypertension** |  |  |  |  |  |  |
| No | 67.5 | 11,61,631 | 71.5 | 2,72,872 | 67.9 | 14,34,503 |
| Yes | 19.8 | 3,40,486 | 18.9 | 77,865 | 19.7 | 4,18,351 |
| Missing | 12.7 | 1,99,072 | 9.6 | 25,998 | 12.4 | 2,25,070 |
| **Consumption of Alcohol** | |  |  |  |  |  |
| No | 91.1 | 15,30,810 | 80.8 | 2,89,646 | 90.1 | 18,20,456 |
| Yes | 8.9 | 1,70,379 | 19.2 | 87,089 | 9.9 | 2,57,468 |
| **Consumption of Tobacco** | |  |  |  |  |  |
| No | 78.1 | 13,06,138 | 65.5 | 2,33,672 | 76.9 | 15,39,810 |
| Yes | 22.0 | 3,95,051 | 34.5 | 1,43,063 | 23.1 | 5,38,114 |
| Overall | 100.0 | 1701189 | 100 | 3,76,735 | 100 | 20,77,924 |

Note: NFHS National Family Health Survey, ST Schedule Tribe

**Supplementary Table 3: Sample description of the study population aged 15 and above in different combinations of districts with concentration of STs in India, NFHS-5 (2019­­–2021)**

|  | **SCHEDULED** | |  |  |  | **NON-SCHEDULED** | |  |  |  |  |  |  |  |
| --- | --- | --- | --- | --- | --- | --- | --- | --- | --- | --- | --- | --- | --- | --- |
|  | Districts marked with Schedule V & VI | | | |  | Districts with >60% ST | |  |  |  | Districts with<60% ST | |  |  |
| Number of Districts (N) | 136 (386,262) | |  |  |  | 51 (132,494) | |  |  |  | 520 (1,559,168) | |  |  |
| Ethnicity | Non-ST |  | ST |  |  | Non-ST |  | ST |  |  | Non-ST |  | ST |  |
| Characteristics | % (weighted) | N (Unweighted) | % (weighted) | N (Unweighted) |  | % (weighted) | N (Unweighted) | % (weighted) | N (Unweighted) |  | % (weighted) | N (Unweighted) | % (weighted) | N (Unweighted) |
| **Age** |  |  |  |  |  |  |  |  |  |  |  |  |  |  |
| 15-29 | 33.4 | 77,564 | 37.9 | 60,328 |  | 36.7 | 5,239 | 35.67 | 40,031 |  | 35.7 | 5,19,666 | 37.6 | 37,162 |
| 30-44 | 28.7 | 64,004 | 28.0 | 45,224 |  | 29.6 | 4,591 | 28.22 | 34,042 |  | 27.1 | 3,97,773 | 27.9 | 28,337 |
| 45-59 | 22.1 | 50,404 | 20.6 | 32,991 |  | 22.1 | 3,324 | 22.84 | 27,891 |  | 21.0 | 3,05,699 | 20.2 | 20,442 |
| 60+ | 15.9 | 35,605 | 13.5 | 20,142 |  | 11.7 | 1,417 | 13.27 | 15,959 |  | 16.2 | 2,35,903 | 14.4 | 14,186 |
| **Sex** |  |  |  |  |  |  |  |  |  |  |  |  |  |  |
| Female | 50.7 | 1,15,890 | 51.6 | 82,071 |  | 49.3 | 7,013 | 51.05 | 59,966 |  | 51.5 | 7,48,149 | 51.5 | 51,214 |
| Male | 49.3 | 1,11,687 | 48.4 | 76,614 |  | 50.7 | 7,558 | 48.95 | 57,957 |  | 48.5 | 7,10,892 | 48.5 | 48,913 |
| **Education** |  |  |  |  |  |  |  |  |  |  |  |  |  |  |
| Illiterate | 22.6 | 54,708 | 37.8 | 56,639 |  | 24.7 | 3,280 | 24.94 | 25,852 |  | 23.8 | 3,62,483 | 35.0 | 33,911 |
| Primary | 14.1 | 33,703 | 15.3 | 25,035 |  | 14.6 | 2,138 | 14.79 | 17,732 |  | 13.5 | 1,89,379 | 14.2 | 14,033 |
| Secondary | 47.5 | 1,09,264 | 40.6 | 67,169 |  | 49.4 | 7,465 | 50.14 | 61,368 |  | 47.3 | 6,95,837 | 42.0 | 43,548 |
| Higher | 15.8 | 29,902 | 6.4 | 9,842 |  | 11.3 | 1,688 | 10.14 | 12,971 |  | 15.5 | 2,11,342 | 8.9 | 8,635 |
| **Wealth Quintile** | |  |  |  |  |  |  |  |  |  |  |  |  |  |
| Poorest | 12.0 | 44,412 | 52.5 | 82,608 |  | 31.2 | 3,103 | 34.96 | 32,617 |  | 16.1 | 2,26,454 | 34.7 | 33,543 |
| Poorer | 17.5 | 47,703 | 24.6 | 40,209 |  | 24.0 | 3,435 | 26.81 | 35,515 |  | 19.1 | 2,94,382 | 24.9 | 27,313 |
| Middle | 22.5 | 48,904 | 12.6 | 20,826 |  | 18.3 | 3,839 | 17.61 | 25,026 |  | 20.4 | 3,09,494 | 19.3 | 20,192 |
| Richer | 24.8 | 47,616 | 6.4 | 10,504 |  | 15.7 | 3,031 | 13.01 | 17,356 |  | 21.4 | 3,09,351 | 12.7 | 12,700 |
| Richest | 23.3 | 38,942 | 3.9 | 4,538 |  | 10.8 | 1,163 | 7.61 | 7,409 |  | 23.0 | 3,19,360 | 8.5 | 6,379 |
| **Place of Residence** | |  |  |  |  |  |  |  |  |  |  |  |  |  |
| Urban | 37.5 | 64,322 | 10.1 | 13,709 |  | 27.1 | 5,153 | 20.42 | 23,994 |  | 34.6 | 4,04,917 | 18.1 | 13,099 |
| Rural | 62.5 | 1,63,255 | 89.9 | 1,44,976 |  | 73.0 | 9,418 | 79.58 | 93,929 |  | 65.4 | 10,54,124 | 81.9 | 87,028 |
| **Household Size** |  |  |  |  |  |  |  |  |  |  |  |  |  |  |
| 1-3 Members | 25.4 | 53,157 | 20.81 | 33,202 |  | 25.7 | 4,633 | 26.5 | 36,371 |  | 21.2 | 2,95,111 | 21.6 | 21315.00 |
| 4-5 Members | 43.0 | 96,929 | 40.55 | 64,916 |  | 42.3 | 6,420 | 40.87 | 50,104 |  | 39.6 | 5,82,341 | 42.0 | 42740.00 |
| 6 & above Members | 31.6 | 77,491 | 38.64 | 60,567 |  | 32.1 | 3,518 | 32.64 | 31,448 |  | 39.3 | 5,81,589 | 36.5 | 36,072 |
| **Persons Sleeping per Room** | | |  |  |  |  |  |  |  |  |  |  |  |  |
| <3 person | 82.2 | 1,71,993 | 78.64 | 1,12,640 |  | 64.3 | 8,881 | 55.39 | 57,836 |  | 72.6 | 10,28,624 | 78.1 | 73,064 |
| 3-4 person | 16.1 | 49,689 | 19.33 | 41,197 |  | 31.5 | 5,049 | 39.57 | 53,073 |  | 24.3 | 3,81,792 | 20.0 | 24358.00 |
| >5 person | 1.7 | 5,895 | 2.03 | 4,848 |  | 4.2 | 641 | 5.04 | 7,014 |  | 3.1 | 48,625 | 2.0 | 2,705 |
| **Type of House** | |  |  |  |  |  |  |  |  |  |  |  |  |  |
| Natural | 16.3 | 58,174 | 52.5 | 86,140 |  | 48.5 | 6,263 | 46.1 | 55,691 |  | 16.2 | 2,62,612 | 35.9 | 39,159 |
| Rudimentary | 16.7 | 38,571 | 17.7 | 27,667 |  | 16.1 | 2,520 | 22.1 | 26,939 |  | 26.0 | 3,77,108 | 23.9 | 23,057 |
| Finished | 67.0 | 1,30,832 | 29.8 | 44,878 |  | 35.4 | 5,788 | 31.81 | 35,293 |  | 57.8 | 8,19,321 | 40.2 | 37,911 |
| **Separate Kitchen** | |  |  |  |  |  |  |  |  |  |  |  |  |  |
| Yes | 65.3 | 1,35,072 | 47.61 | 78,329 |  | 64.3 | 9,466 | 53.63 | 60,690 |  | 58.0 | 8,48,279 | 50.5 | 50,646 |
| No | 18.1 | 42,294 | 28.12 | 39,880 |  | 18.9 | 2,620 | 21.53 | 26,329 |  | 22.8 | 3,15,871 | 24.0 | 23,110 |
| Missing | 16.6 | 50,211 | 24.27 | 40,476 |  | 16.8 | 2,485 | 24.84 | 30,904 |  | 19.2 | 2,94,891 | 25.5 | 26,371 |
| **Type of Fuel Used for Cooking** | | |  |  |  |  |  |  |  |  |  |  |  |  |
| Improved | 69.8 | 1,28,405 | 25.1 | 37,608 |  | 45.7 | 9,136 | 38.37 | 51,542 |  | 59.1 | 8,27,204 | 41.2 | 39,929 |
| Unimproved | 30.2 | 99,172 | 74.9 | 1,21,077 |  | 54.3 | 5,435 | 61.63 | 66,381 |  | 40.9 | 6,31,837 | 58.8 | 60,198 |
| **Potability of Water** | |  |  |  |  |  |  |  |  |  |  |  |  |  |
| Yes | 54.5 | 1,27,361 | 56.4 | 92,273 |  | 70.3 | 10,903 | 79.3 | 98,232 |  | 38.8 | 5,89,822 | 39.4 | 43,308 |
| No | 45.5 | 1,00,216 | 43.6 | 66,412 |  | 29.7 | 3,668 | 20.7 | 19,691 |  | 61.2 | 8,69,219 | 60.6 | 56,819 |
| **Shared Toilet** |  |  |  |  |  |  |  |  |  |  |  |  |  |  |
| No | 77.7 | 1,72,516 | 57.87 | 1,02,607 |  | 74.7 | 11,802 | 82.13 | 1,06,481 |  | 75.4 | 11,20,260 | 63.0 | 67,862 |
| Yes | 6.6 | 14,744 | 4.36 | 6,636 |  | 7.9 | 1,708 | 5 | 6,844 |  | 8.3 | 1,13,673 | 6.3 | 6,174 |
| Missing | 15.7 | 40,317 | 37.77 | 49,442 |  | 17.4 | 1,061 | 12.86 | 4,598 |  | 16.3 | 2,25,108 | 30.7 | 26,091 |
| **Smoking Inside the House** | | |  |  |  |  |  |  |  |  |  |  |  |  |
| Never | 64.3 | 1,29,854 | 46.0 | 64,422 |  | 48.1 | 6,228 | 32.23 | 35,500 |  | 56.9 | 7,92,679 | 48.9 | 44,531 |
| Yes | 35.7 | 97,723 | 54.0 | 94,263 |  | 51.9 | 8,343 | 67.77 | 82,423 |  | 43.1 | 6,66,350 | 51.1 | 55,596 |
| **Diabetes** |  |  |  |  |  |  |  |  |  |  |  |  |  |  |
| No | 74.8 | 1,75,146 | 79.82 | 1,29,840 |  | 79.5 | 11,898 | 83.23 | 99,084 |  | 73.5 | 10,97,230 | 78.7 | 79,727 |
| Yes | 12.1 | 25,858 | 8.68 | 13,790 |  | 10.9 | 1,543 | 9.35 | 11,782 |  | 11.6 | 1,57,199 | 9.4 | 9,082 |
| Missing | 13.2 | 26,573 | 11.5 | 15,055 |  | 9.6 | 1,130 | 7.42 | 7,057 |  | 14.9 | 2,04,612 | 11.9 | 11,318 |
| **Hypertension** |  |  |  |  |  |  |  |  |  |  |  |  |  |  |
| No | 67.4 | 1,58,282 | 71.48 | 1,16,577 |  | 70.2 | 10,122 | 72.82 | 84,696 |  | 67.5 | 9,93,227 | 71.3 | 71,599 |
| Yes | 21.4 | 47,276 | 18.9 | 29,830 |  | 22.1 | 3,617 | 21.86 | 29,032 |  | 19.5 | 2,89,593 | 18.5 | 19,003 |
| Missing | 11.2 | 22,019 | 9.62 | 12,278 |  | 7.8 | 832 | 5.33 | 4,195 |  | 12.9 | 1,76,221 | 10.2 | 9,525 |
| **Consumption of Alcohol** | |  |  |  |  |  |  |  |  |  |  |  |  |  |
| No | 90.5 | 2,02,959 | 78.9 | 1,21,159 |  | 83.4 | 11,399 | 78.65 | 89,057 |  | 91.2 | 13,16,452 | 83.2 | 79,430 |
| Yes | 9.5 | 24,618 | 21.1 | 37,526 |  | 16.6 | 3,172 | 21.35 | 28,866 |  | 8.8 | 1,42,589 | 16.8 | 20,697 |
| **Consumption of Tobacco** | |  |  |  |  |  |  |  |  |  |  |  |  |  |
| No | 79.0 | 1,70,117 | 63.2 | 96,104 |  | 69.0 | 9,587 | 57.82 | 69,947 |  | 77.9 | 11,26,434 | 69.1 | 67,621 |
| Yes | 21.0 | 57,460 | 36.8 | 62,581 |  | 31.0 | 4,984 | 42.18 | 47,976 |  | 22.1 | 3,32,607 | 30.9 | 32,506 |
| **Overall** | **15.8** | **227577** | **51.8** | **1,58,685** |  | **0.1** | **14,571** | **5.08** | **1,17,923** |  | **84.1** | **14,59,041** | **43.2** | **1,00,127** |

Note: NFHS National Family Health Survey, ST Schedule Tribe
